# Neutrophils to Lymphocyte Ratio as a Biomarker in Bronchiectasis Exacerbation: A Retrospective Study

**DOI:** 10.7759/cureus.9728

**Published:** 2020-08-13

**Authors:** Vasiliki E Georgakopoulou, Nikolaos Trakas, Christos Damaskos, Nikolaos Garmpis, Evgenia Karakou, Rea Chatzikyriakou, Panagiota Lambrou, Xanthi Tsiafaki

**Affiliations:** 1 Department of Pulmonology, Laiko General Hospital, Athens, GRC; 2 1st Department of Pulmonology, Sismanogleio Hospital, Athens, GRC; 3 Department of Biochemistry, Sismanogleio Hospital, Athens, GRC; 4 Renal Transplantation Unit, Laiko General Hospital, Athens, GRC; 5 Laboratory of Experimental Surgery and Surgical Research "N.S. Christeas", National and Kapodistrian University of Athens School of Medicine, Athens, GRC; 6 2nd Department of Propedeutic Surgery, Laiko General Hospital, Athens, GRC; 7 Department of Hematology, Sismanogleio Hospital, Athens, GRC

**Keywords:** bronchiectasis exacerbation, sputum cultures, neutrophil to lymphocyte ratio (nlr)

## Abstract

Introduction

Bronchiectasis is a disorder resulting mainly from bronchial inflammation caused by recurrent or chronic infections. It is characterized by permanently dilated airways due to bronchial wall destruction. Exacerbations have a key role in bronchiectasis as they are associated with a negative impact on patient prognosis. Exacerbations are generally infectious events caused mostly by bacterial microorganisms. Infective or inflammatory agents cause neutrophil recruitment into the airways, which leads to proteolytic enzymes such as neutrophil elastase and matrix metalloproteinases release, resulting in airway matrix destruction. Neutrophil to lymphocyte ratio (NLR) is used as a biomarker of inflammation. It is calculated by dividing the number of neutrophils by the number of lymphocytes. Our aim is to evaluate Neutrophils to Lymphocyte Ratio in patients with bronchiectasis exacerbation and its correlation to microbiological data.

Methods

The study involved patients with a diagnosis of bronchiectasis based on high-resolution computerised tomography (HRCT) of the chest who fulfilled the criteria of bronchiectasis exacerbation. Complete blood counts with differential counts, which included total white blood cells, neutrophils and lymphocytes, were obtained. NLR and C-reactive protein (CRP) levels were measured in patients with bronchiectasis exacerbation and in healthy controls. NLR was calculated as the ratio of the neutrophils to lymphocytes. The mean NLR values in patients with bronchiectasis exacerbation were compared to mean NLR values in healthy controls. The NLR values were compared to CRP levels in patients with bronchiectasis exacerbation. Sputum cultures were performed in all patients. The mean NLR values in patients with positive sputum cultures were compared with mean NLR values in patients with negative sputum cultures, and mean NLR values in patients with isolated Pseudomonas aeruginosa in sputum cultures were compared to mean NLR values in patients with other infectious agents isolated.

Results

The study population consisted of 80 patients with bronchiectasis exacerbation - 54 males and 26 females - with a mean age of 77.3±8.4 years, and 64 healthy controls - 36 males and 28 females - with a mean age of 62.9±15.3 years. The mean CRP levels in patients with bronchiectasis exacerbation were 75.03±73.87 mg/l. The mean NLR value in patients with bronchiectasis exacerbation was 9.2±7.8 and the mean NLR value of controls was 3.1±2.9 (p<0.001). The NLR values in patients with bronchiectasis exacerbation had no linear correlation with CRP values in these patients (r=0.002, p=0.992). Fifty-two patients had positive sputum cultures and 28 patients had negative sputum cultures. The mean NLR value in patients with positive sputum cultures was 10.5±9.1, and in patients with negative sputum cultures, it was 6.7±3.6 (p<0.012). The mean NLR value in patients with P.aeruginosa was 10.1±9.5, and in patients with other microorganisms isolated, it was 10.8±8.9 (p=0.784).

Conclusions

Neutrophil to lymphocyte ratio values are statistically greater in patients with bronchiectasis exacerbation compared to healthy controls. There is no linear correlation between NLR and CRP in these patients. NLR values are statistically greater in patients with positive sputum cultures compared to those with negative sputum cultures. Therefore, NLR can be used for predicting positive cultures in patients with bronchiectasis exacerbation.

## Introduction

Non-cystic fibrosis bronchiectasis is a disorder resulting mainly from bronchial inflammation caused by recurrent or chronic infections. This disease is characterized by permanently dilated airways due to bronchial wall destruction. Clinical features of bronchiectasis are chronic cough, hemoptysis, impaired mucus clearance resulting in increased sputum production, dyspnea and frequent bacterial colonization and infections [1]. The most common causes of bronchiectasis are postinfective, chronic obstructive pulmonary disease (COPD), connective tissue diseases, immunodeficiency, asthma, allergic bronchopulmonary aspergillosis, ciliary dysfunction, inflammatory bowel disease, aspiration/esophageal reflux, congenital malformation, α1-antitrypsin deficiency, yellow nail syndrome and idiopathic [2].

Several definitions of bronchiectasis exacerbation have been described. Definition of bronchiectasis exacerbation has been reported as a person with known bronchiectasis with a deterioration in three or more of the following symptoms for at least 48 hours: cough, sputum volume and/or consistency, sputum purulence, breathlessness or exercise tolerance, fatigue and/or malaise, hemoptysis and a clinician determining that a change in bronchiectasis treatment is needed [3]. According to the Spanish Guidelines on Treatment of Bronchiectasis in Adults, bronchiectasis exacerbation is defined as a clinical condition in a person with known bronchiectasis and increasing cough and changes in sputum characteristics (increased volume, thicker consistency, greater purulence), which may be accompanied by worsening dyspnea, fever, asthenia, general discomfort, anorexia, weight loss, chest pain, hemoptysis, changes in thoracic objective exam, requirement of changes in bronchiectasis treatment and declining lung function. These guidelines also mentioned the term "very severe exacerbations" characterized by hemodynamic instability, altered mental status or the need for admission to the intensive care unit [4].

Exacerbations have a key role in bronchiectasis as they are associated with a negative impact on patient prognosis. Increased frequency of exacerbations leads to increased airway inflammation, progressive lung damage, accelerated lung function decline and increased mortality. Exacerbations are generally infectious events caused mostly by bacterial microorganisms. Comorbidities, such as gastroesophageal reflux, are considered potential causes of bronchiectasis exacerbations [5]. Several microorganisms are associated with bronchiectasis. Pseudomonas aeruginosa (P. aeruginosa) is one of the most common and important microorganisms in patients with bronchiectasis, and its presence is related to greater impairment in lung function, more frequent exacerbations, worse quality of life, greater risk of hospitalisation and mortality [6].

Neutrophils have a major role in the development and progression of bronchiectasis. In biopsies from bronchial mucosa in patients with bronchiectasis, tissue neutrophilia and mononuclear cell infiltrate have been noticed [7]. Infective or inflammatory agents cause neutrophil recruitment into the airways, which leads to proteolytic enzymes such as neutrophil elastase and matrix metalloproteinases release, resulting in airway matrix destruction [8]. The destruction of the epithelial layer leads to reduced mucociliary clearance, which is responsible for bacterial colonization and preserving the vicious circle of ''microbial load-inflammation response lung damage-impaired bronchial clearance''. In addition, increased blood neutrophils have been reported in patients with bronchiectasis, with correlation with disease severity and bacterial colonization [9].

Neutrophil to lymphocyte ratio (NLR) is used as a biomarker of inflammation. It is calculated by dividing the number of neutrophils by the number of lymphocytes, usually from a peripheral blood sample [10]. This marker has been evaluated in several clinical conditions. A recent meta-analysis study reported that a high NLR is an independent factor associated with worse overall survival in several solid tumors [11]. NLR has been explored as an index of systemic inflammation that predicts prognosis in adults with community-acquired pneumonia (CAP) [12]. In addition, NLR has been investigated as an infectious exacerbation marker in patients with COPD and chronic respiratory failure [13] and may be related to renal or hepatic dysfunction, diabetes mellitus, thyroid disorders, hypertension, metabolic syndrome, hematological malignancies and other inflammatory diseases [14].

Our aim is to evaluate the neutrophil to lymphocyte ratio in patients with bronchiectasis exacerbation and its correlation to microbiological data.

## Materials and methods

This is a retrospective observational study. The study involved patients with a previous diagnosis of bronchiectasis based on high-resolution computerised tomography (HRCT) of the chest who fulfilled the criteria of bronchiectasis exacerbation [3] and were hospitalised between 1 January 2016 and 1 January 2019 at the 1st Pulmonary Department in Sismanogleio Hospital in Athens and at the 1st Department of Internal Medicine in Laiko General Hospital in Athens. The exclusion criteria were: patients <18 years old because we wanted to evaluate this marker in adults; patients receiving immunosuppressive therapy; patients with myeloproliferative disorders; and patients with hematological malignancies and solid tumors. In addition, age- and sex-matched healthy controls without infection were involved. Whole blood samples were collected from healthy controls and from the patients on admission. Healthy controls were people who had visited the health care settings for a routine check-up without underlying disease, without symptoms and signs of infection and with C-reactive protein (CRP) values less than 6 mg/l. Verbal consent was obtained from all the participants. Complete blood counts with differential counts, which included total white blood cells, neutrophils and lymphocytes, were obtained from automated ΧΕ 2100 hematology analyzer (Sysmex Corporation, Kobe, Japan). NLR and CRP levels were measured in patients with bronchiectasis exacerbation and in healthy controls. NLR was calculated as the ratio of the neutrophils to lymphocytes. Serum levels of CRP were determined by immunoturbidimetric assay on Cobas 6000 c501 analyzer (Roche, Basel, Switzerland). Normal values for CRP are values less than 6 mg/l. The mean NLR values in patients with bronchiectasis exacerbation were compared to mean NLR values in healthy controls. The NLR values were compared to CRP levels in patients with bronchiectasis exacerbation. Additionally, sputum cultures were performed in all patients. The mean NLR values in patients with positive sputum cultures were compared with mean NLR values in patients with negative sputum cultures, and the mean NLR values in patients with isolated P. aeruginosa in sputum cultures were compared to mean NLR values in patients with other infectious agents isolated. IBM SPSS® version 19.0 was used for the statistical analysis of data. Initially, normality of data was examined by using the Kolmogorov-Smirnov (KS) test. Independent samples t-test was performed for the analysis of normal data. The results were presented as mean±standard deviation (variables with normal distribution). A p-value of less than 0.05 (p <0.05) was considered statistically significant.

## Results

The study population consisted of 80 patients with bronchiectasis exacerbation - 54 males and 26 females - with a mean age of 77.3±8.4 years, and 64 healthy controls - 36 males and 28 females - with a mean age of 62.9±15.3 years (Table 1).

**Table 1:** Characteristics of the study population C: controls; P: patients with bronchiectasis exacerbation; NLR: neutrophil to lymphocyte ratio

	C n=64	P n=80	C versus P
Gender (males/females)	36/28	54/26	0.166
Age(years) (SD)	62.9(15.3)	77.3(8.4)	<0.001
NLR (SD)	3.1(2.9)	9.2(7.8)	<0.001

**Figure 1:**
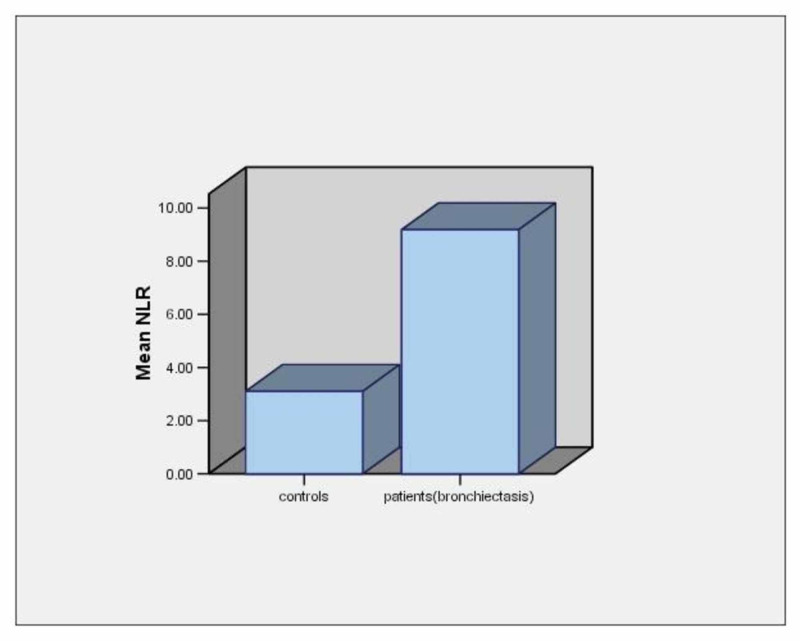
Mean NLR values in patients with bronchiectasis exacerbation and healthy controls NLR: neutrophil to lymphocyte ratio

The mean CRP levels in patients with bronchiectasis exacerbation were 75.03±73.87 mg/l. The mean NLR value in patients with bronchiectasis exacerbation was 9.2±7.8 and the mean NLR value of controls was 3.1±2.9 (p<0.001) (Table 1, Figure 1). 

**Figure 2:**
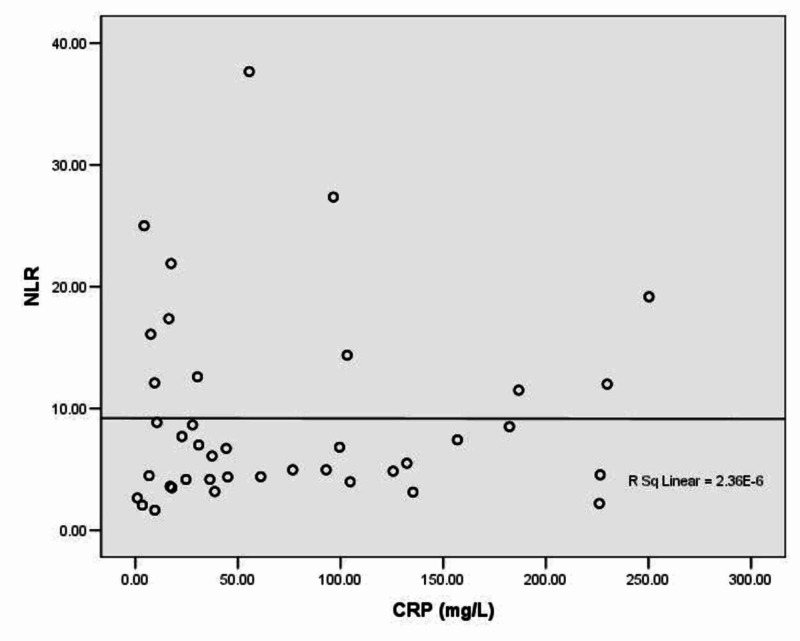
Correlation between NLR and CRP values in patients with bronchiectasis exacerbation NLR: neutrophil to lymphocyte ratio; CRP: C-reactive protein

The NLR values in patients with bronchiectasis exacerbation had no linear correlation with CRP values in these patients (r=0.002, p=0.992) (Figure 2). 

**Figure 3:**
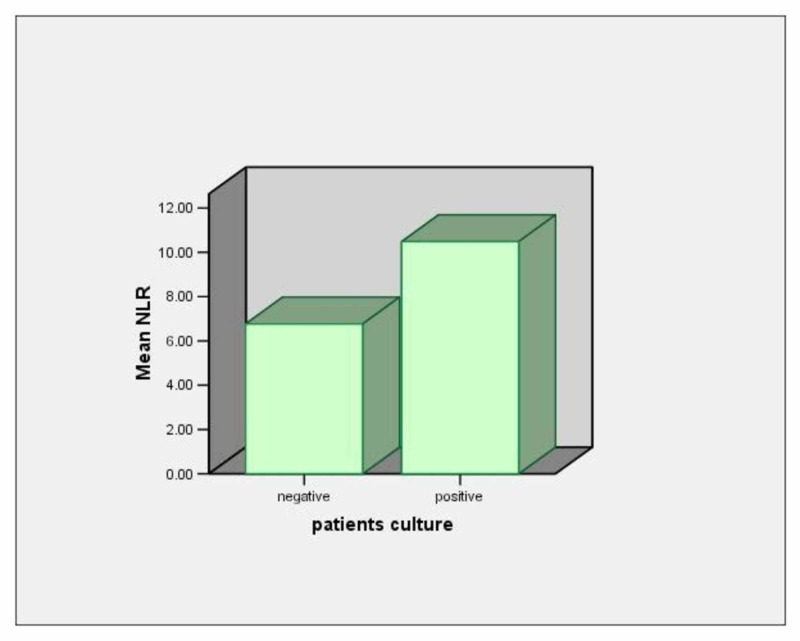
Mean NLR values in patients with positive and negative sputum cultures NLR: neutrophil to lymphocyte ratio

Fifty-two patients had positive sputum cultures and 28 patients had negative sputum cultures. The mean NLR value in patients with positive sputum cultures was 10.5±9.1 and in patients with negative sputum cultures was 6.7±3.6 with p<0.012 (Table 2, Figure 3). 

**Table 2:** NLR in patients with positive and negative sputum cultures NLR: neutrophil to lymphocyte ratio

Patients culture	positive n=52	negative n=28	positive versus negative
NLR (SD)	10.5(9.1)	6.7(3.6)	<0.012

**Figure 4:**
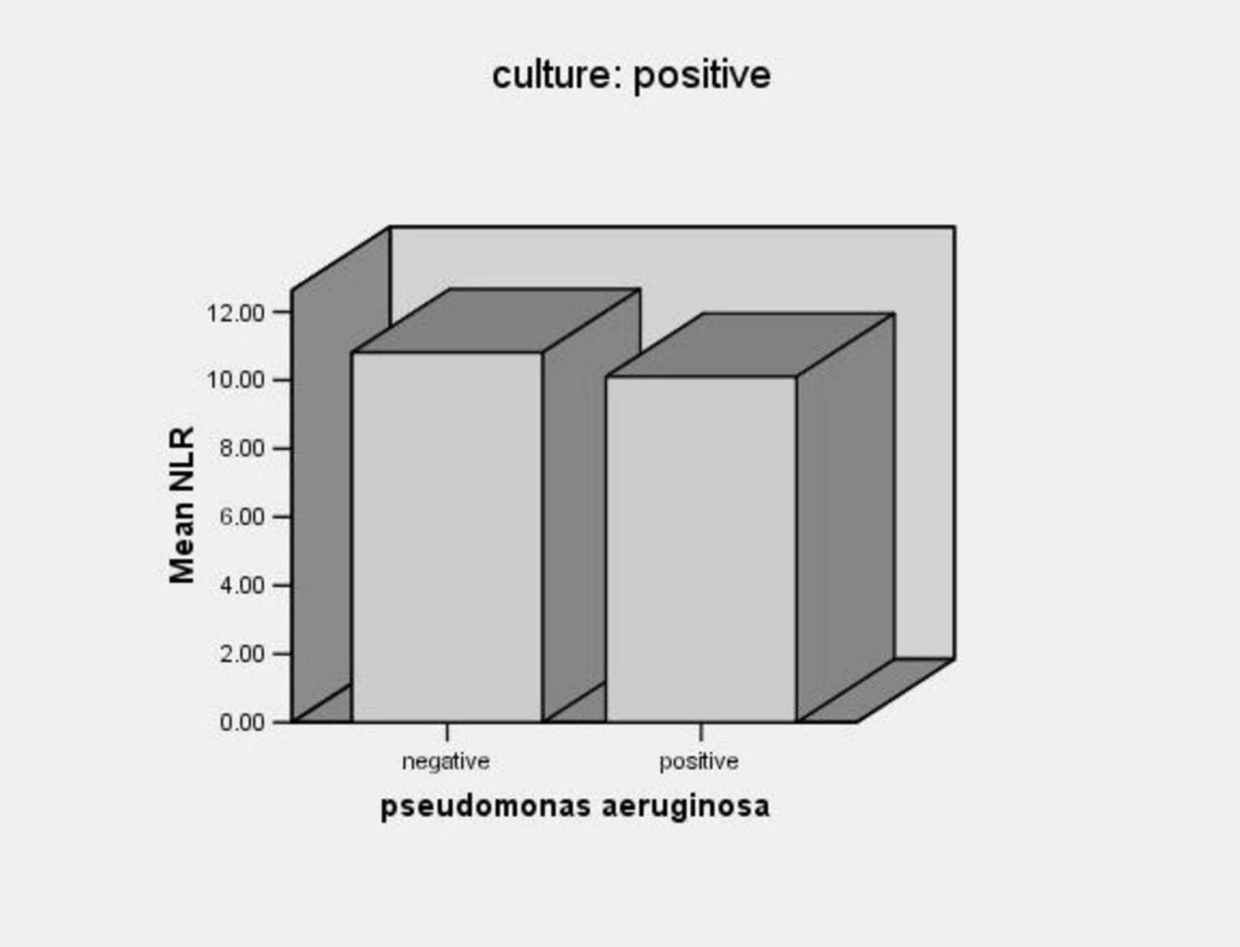
Mean NLR values in patients with Pseudomonas aeruginosa and patients with other microorganism isolated NLR: neutrophil to lymphocyte ratio

The mean NLR value in patients with P. aeruginosa was 10.1±9.5, and in patients with other microorganisms isolated it was 10.8±8.9 with p=0.784 (Table 3, Figure 4).

**Table 3:** Mean NLR values in patients with Pseudomonas aeruginosa and patients with other microorganism isolated *P.A: Pseudomonas aeruginosa; NLR: neutrophil to lymphocyte ratio

Patients culture positive	P.A (pos) n=24	Other Microorganism n=28	positive versus negative
NLR (SD)	10.1(9.5)	10.8(8.9)	0.784

## Discussion

According to our results there was a statistically significant difference in mean NLR value between patients with bronchiectasis exacerbation and healthy controls. NLR reflects the state of balance between neutrophils and lymphocytes. The higher NLR is associated with more inflammatory response [15]. NLR has been used as a biomarker in numerous diseases because the physiological response of circulating leukocytes to inflammation is an increase in circulating neutrophils and a decrease in the number of lymphocytes [16].

To our knowledge, the current study is the first to evaluate NLR in bronchiectasis exacerbation in adults. Nacaroglu et al. evaluated the role of NLR in bronchiectasis exacerbation in children and found leukocyte count, absolute neutrophil count and NLR in children with bronchiectasis exacerbation were higher when compared to the control group [17]. Coban et al. investigated, for the first time, the role of NLR in patients with stable bronchiectasis. They studied the correlation between NLR ratio and FACED (FEV1% predicted [**F**orced expiratory volume in first second], **A**ge, **C**hronic colonization by P. aeruginosa, radiological **E**xtension of the disease, **D**yspnea) or BSI (Bronchiectasis Severity Index) scores, which are used for the assessment of the severity of bronchiectasis in patients with stable bronchiectasis. From their study, there was no significant correlation between NLR ratio and FACED or BSI scores in patients with stable bronchiectasis [18]. Bedi et al. described that systemic neutrophils from patients with stable bronchiectasis have a higher level of activation compared with healthy controls, and blood neutrophil viability is significantly prolonged because of delayed apoptosis (a feature of inflammation) [19].

In our study, there was no linear correlation between NLR and CRP values in patients with bronchiectasis exacerbation. C-reactive protein has been evaluated in patients with stable bronchiectasis. Hsieh et al. demonstrated a good correlation between serum CRP and HRCT scores in the patients with stable bronchiectasis [20], while Posadas et al. reported that the CRP value was related to a greater risk of future severe exacerbations in patients with stable bronchiectasis [21]. Nacaroglu et al. found a moderately significant, positive correlation between NLR and acute exacerbation period CRP levels (p<0.001) in children [17].

Additionally, in our study, there was a statistically significant difference in the mean NLR value between patients with positive sputum cultures and patients with negative sputum cultures, indicating that NLR can be used to predict positive sputum cultures. Positive sputum cultures have been associated with significantly higher mortality in patients with stable bronchiectasis compared with non-colonized patients, according to a study by Chalmers et al. [22]. In the same study, the mortality rate varied significantly depending on the isolated organism, with the highest mortality associated with the isolation of P. aeruginosa. As mentioned in the introduction, P.aeruginosa is related to greater impairment in lung function, more frequent exacerbations, worse quality of life, greater risk of hospitalisation and mortality [6]. However, mean NLR values in patients with isolation of P.aeruginosa did not have statistically significant difference compared to mean NLR values in patients with isolation of other microorganisms.

NLR has been evaluated in other respiratory diseases. Furukawa et al. showed that the number of neutrophils in sputum were increased in children with asthma and there was a small correlation between the NLR with the number of hospital admissions [23]. Gunay et al. reported that NLR values were higher than controls in patients with stable COPD, and there was a positive relationship between CRP and NLR in these patients [24].

There were some limitations. It was a retrospective study from two centers, with relatively small sample size of patients that does not permit to generalize the results. A further well-controlled, multicenter prospective study is needed to clarify the role of NLR in patients with bronchiectasis exacerbation.

## Conclusions

Neutrophil to lymphocytes ratio values are statistically greater in patients with bronchiectasis exacerbation compared to healthy controls. Further investigation is needed to establish the diagnostic significance of this marker in patients with bronchiectasis exacerbation - maybe a study comparing NLR values between patients with stable bronchiectasis and patients with exacerbation. There is no linear correlation between NLR and CRP in these patients. NLR values are statistically greater in patients with positive sputum cultures compared to those with negative sputum cultures. Therefore, NLR can be used for predicting positive cultures in patients with bronchiectasis exacerbation, and the presence of positive cultures can change the initial antibiotic therapy according to isolated microorganism and antimicrobial susceptibility testing.
